# Non-home discharge in the octogenarian and nonagenarian colorectal cancer population: a retrospective cohort study

**DOI:** 10.1007/s00384-025-04891-0

**Published:** 2025-04-21

**Authors:** Madhu Bhamidipaty, Janani Thillainadesan, Matthew Rickard, Anil Keshava, Vincent Lam, Michael Suen

**Affiliations:** 1 Department of Colorectal Surgery, Macquarie University Hospital, Macquarie University, Sydney, NSW Australia; 2 Macquarie Medical School, Faculty of Medicine, Health and Human Sciences, Macquarie University, Sydney, NSW Australia; 3 Concord Institute of Academic Surgery, Concord Repatriation General Hospital, Sydney, NSW Australia; 4 Discipline of Surgery, School of Medicine, Faculty of Medicine and Health, University of Sydney, Sydney, NSW Australia; 5 Division of Colorectal Surgery, Department of Surgery, Concord Repatriation General Hospital, Sydney, NSW Australia; 6 Concord Clinical School, Faculty of Medicine and Health, University of Sydney, Sydney, NSW Australia; 7 Department of Colorectal Surgery, Launceston General Hospital, Launceston, TAS, Australia

**Keywords:** Colorectal cancer, Elderly, Non-home discharge, Discharge disposition, Geriatric

## Abstract

**Purpose:**

This study aims to determine the rate of non-home discharge (NHD) and identify factors associated with non-home discharge in a colorectal cancer (CRC) population of adults aged 80 years and older. This is the first study looking specifically at NHD as an outcome in the ≥ 80 years colorectal cancer cohort.

**Methods:**

This is a single-centre retrospective exploratory observational study from a high-volume colorectal cancer unit. Patients aged ≥ 80 years from a prospectively collected CRC database from 2013 to 2020 were included. Electronic medical records were assessed to obtain demographic, clinical, functional and discharge data. Univariable and multivariable logistic regression analyses were performed to identify factors associated with NHD the primary study outcome. Secondary outcomes included discharge disposition and functional decline.

**Results:**

Two hundred forty-two patients aged ≥ 80 years underwent CRC resection. Alo, 234 patients and 221 patients were included in the overall and subgroup non-home discharge analysis. The non-home discharge rate was 19.9% in the cohort that pre-operatively were from home. On multivariable logistic regression, after adjusting for other significant variables, frailty (odds ratio (OR) 2.91, 95% CI 1.25–6.75, *p* = 0.013), severe complications (OR 3.92, 95% CI 1.40–10.97, *p* = 0.009) and an open operation (OR 3.93, 95% CI 1.87–8.24, *p* < 0.001) were associated with a significantly higher rate of NHD. The incidence of functional decline from those at home was 72.4% in the non-home discharge group and 16.7% in those who returned home (*p* < 0.001).

**Conclusion:**

This is the first paper describing the overall rate and identifying factors associated with non-home discharge specifically in the ≥ 80 years CRC population. Prospective studies are required to investigate causality and interventions to reduce non-home discharge rates.

## Introduction

Colorectal cancer (CRC) is the third most common cancer globally and has a high incidence in older adults [1, 2]. This population are more frail, comorbid, lack the physiological reserve to tolerate complications and are less likely to be surgical candidates [3]. Observational studies have shown that octo- and nonagenarians are less likely to be discharged directly home and instead are transferred to a geriatric, rehabilitation or aged care facility after their operative admission [4, 5]. The definition and practice of these discharge facilities vary across countries and health care systems.

Whilst increased age is associated with greater postoperative morbidity, emerging evidence suggests that older adults can benefit from a curative resection, thus age alone should not be a contraindication for offering surgery [6]. The evaluation of outcomes that are highly valued by older adults has been highlighted as a key gap in surgical intervention trials [7]. These outcomes include quality of life, functional and cognitive recovery, and return to home. It has been observed in other specialties that the patient’s ability to return to their home after an operation helps their biopsychosocial recovery [8, 9]. Outcomes such as ‘non-home discharge’ (NHD) are being evaluated in geriatric, gynaecological, vascular, cardiac and orthopaedic literature [9, 10] as it can influence operative decision-making in this elderly population. With significant improvements in modern healthcare and a greater life expectancy, outcomes such as NHD are becoming increasingly relevant for colorectal surgeons [1]. To date, few studies have examined the outcome of NHD in colorectal patients, but none studied specifically the octogenarian CRC cohort. This study investigates this important functional and increasingly relevant outcome in the Australian octogenarian CRC population. The aim of this paper was to determine the NHD rate in an Australian population and identify factors associated with higher rates of NHD in this elderly CRC cohort.

## Materials and methods

### Study design, participants and setting

This is a retrospective observational exploratory study utilising prospectively collected data from an Australian CRC centre. Patients were identified from the Concord Hospital CRC database. Data was stored on the online, password-protected and secure REDCap Sydney Local Health District (SLHD) database.

Inclusion criteria included all consecutive patients aged ≥ 80 who underwent a curative colorectal cancer resection from 2013 to 2020. This included all patients undergoing elective or emergency surgery. Exclusion criteria included patients who had their surgery in another hospital, palliative-based diversion, non-resection operations, non-cancer resections and endoscopic resections/trans-anal minimally invasive surgery for early cancers.

### Study outcomes and variables

#### Primary outcome

The primary outcome was NHD. This was defined as discharge to a skilled nursing facility/skilled aged care facility (SACF), home with full-time carers, inpatient rehabilitation unit (including both within the same hospital or external facility) and/or a separate acute geriatric or medical unit within the same hospital [9], provided this was different from their pre-admission living arrangements. Death was included in discharge disposition data but excluded from non-home discharge analysis.

A subgroup analysis of those specifically from home and who are independent were analysed, given those from a SACF would only have one outcome, if death were excluded.

#### Secondary outcomes

Secondary outcomes included discharge disposition, functional decline, length of stay and severe complications. Functional decline was measured as a binary; either *‘no* *change or improved activity of daily living (ADL) status’* or *‘decline in ADL status’* excluding those who had died during the operative admission.

#### Other study variables

Baseline demographic variables included age, gender, body mass index (BMI), smoking status, non-age (CCI)- and age-adjusted Charlson Comorbidity Index (ACCI) and admission acuity (elective or emergency). The colorectal unit at Concord Hospital follows an enhanced recovery after surgery (ERAS) protocol for perioperative colorectal care. Those who did not undergo the protocolised ERAS pathway were categorised as ‘non-ERAS’. Elective patients were defined as those being admitted from home. Semi-elective patients are those who may have been admitted acutely or transferred from another medical unit to the colorectal unit for stable symptoms (i.e. per rectal bleeding, anaemia), or incidental finding on imaging that did not necessitate an urgent operation. Semi-elective patients were included in the elective category, given they had time for physiological optimisation. These patients had cancer confirmed on endoscopy and were placed on the next available elective operating list. Emergency patients were defined as those requiring urgent intervention within 72 h, such as clinically obstructing cancers, perforations, or bleeding with haemodynamic instability. Demographic data was analysed as per admission acuity status (elective vs emergency) as it is well established that these groups have different outcomes post-colorectal cancer surgery [11].

Baseline functional variables include frailty and activities of daily living (ADL) status. Frailty was measured using the Rockwood Clinical Frailty Score (CFS), which has been validated to be used in retrospective data analysis [12]. The CFS was stratified into ‘non-frail’ (CFS 1–4) and ‘frail’ (CFS 5–9) as defined previously for elderly CRC patients in the literature [13–15]. Functional status was assessed using the Katz ADL score out of 6 at baseline (pre-operative) and at the time of discharge from hospital (to home, a different hospital or aged care facility).

Severe complications (excluding mortality) were defined as Clavien–Dindo (CD) classification III and IV [16]. CD V (Mortality) was recorded in discharge disposition but excluded from NHD analysis. Length of stay was defined by the difference in the date of the operation (elective or emergency) to the date of discharge from the colorectal unit.

### Statistical analysis

Statistical analysis was performed using IBM SPSS Statistics v28 (IBM Corp, Armonk, NY, USA). Means (standard deviations) and Student’s *t*-test were used for parametric continuous data. Median (interquartile ranges) and Mann–Whitney *U* test were performed for non-parametric continuous data. Proportions were reported as percentages, and differences between proportions were assessed by the chi-squared test.

Confounders, effect modifiers, correlations and collinearity were investigated and adjusted for prior to performing univariable and multivariable regression analysis. After adjustment, only those factors that were significant on univariable analysis were included for the multivariable binary logistic regression analysis. A Hosmer–Lemeshow goodness-of-fit test was used for calibration and a *c*-statistic was obtained from a received operator curve for discrimination. A stepwise backward elimination Wald approach was utilised for the regression analysis, with entry at *p* = 0.05 and a removal at *p* = 0.1. All odds ratios (ORs) were reported with a confidence interval (CI) of 95%. An α-error of 0.05 was used to determine statistical significance.

### Bias minimisation

Minimisation of interpreter bias in retrospective assignment of frailty and functional status was addressed by having two authors independently determine these scores for a sample of patients, one of whom is a practising academic geriatrician.

#### Ethics

This manuscript was prepared using the STROBE guidelines. The protocol was approved by the SLHD Ethics Committee (2021/ETH00864).

## Results

**Fig. 1:**
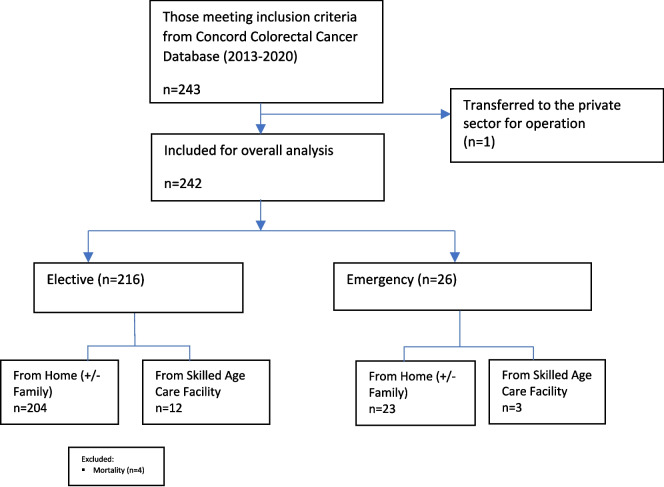
Flowchart on patients included for non-home discharge analysis. NHD, non-home discharge; NH, nursing home; H, home; SACF, skilled aged care facility

In total, 243 patients were identified through the Concord colorectal cancer database who met the inclusion criteria. Of these, one patient was transferred to the private sector and excluded from further analysis. There were 214 octogenarians and 28 nonagenarians, with 216 undergoing an elective operation (Fig. 1).

### Baseline characteristics

The mean age was 85.1 ± 3.8 years (Table 1). The cohort had a male predisposition (54.1%), an average BMI of 26.0 ± 4.7 and a median CCI and ACCI of 4.0 and 8.0, respectively. Of this elderly cohort, 37.2% were smokers or ex-smokers and 42.6% were on an antiplatelet or anticoagulant agent prior to their admission. The operative time was slightly less in the emergency cohort; however, this did not achieve statistical significance. Age aside, there was no significant difference between the emergency and elective patients.

**Table 1:** Overall baseline demographic, functional and operative factors of all patients (*n* = 242), including those excluded from NHD analysis

		**Admission acuity**		
	**Overall (*n* = 242)**	**Elective (*n* = 216)**	**Emergency (*n* = 26)**	***p*-value**
**Baseline factors**				
Age, mean ± SD	85.1 ± 3.75	85.0 ± 3.60	85.9 ± 4.77	***0.035***
Aged 80–90	214	193	21	0.165
	84.1 ± 2.69	84.1 ± 2.66	84.1 ± 3.07	
Aged > 90	28	23	5	0.870
	92.4 ± 2.29	*n* = 23, 92.2 ± 2.20	*n* = 5, 93.5 ± 2.69	
**Gender**				0.975
▪ Male	131 (54.1%)	117 (54.2%)	14 (53.8%)	
▪ Female	111 (45.9%)	99 (45.8%)	12 (46.2%)	
**BMI**	26.0 ± 4.66	26.1 ± 4.66	24.2 ± 4.31	0.565
**Baseline pre-morbid factors**				
CCI (non-age-adjusted) median (IQR)	4.0 (3.0–6.0)	4.0 (3.0–6.0)	4.0 (3.0–6.0)	0.940
CCI (age-adjusted) median (IQR)	8.0 (7.0–10.0)	8.0 (7.0–10.0)	8.0 (7.0–10.0)	0.895
Ex-smoker/smoker (vs non-smoker)	90 (37.2%)	81 (37.5%)	9 (34.6%)	0.774
On an antiplatelet/coagulant (*n* = 242)	103 (42.6%)	89 (41.2%)	14 (53.8%)	0.218
**ERAS pathway**				** < 0.001**
ERAS pathway	167 (69.0%)	162 (75.0%)	5 (19.2%)	
Non-ERAS pathway	75 (31.0%)	54 (25.0%)	21 (80.8%)	
**Operative factors**				
Operation time (min) (*n* = 224)	219.9 ± 72.3	221.6 ± 73.8	202.7 ± 60.8	0.370
**Functional factors**				
Pre-operative living situation (*n* = 242)				*0.232*
▪ Independent living (± family)	227 (93.8%)	204 (94.4%)	23 (88.5%)	
▪ Inpatient/resident rehab/HLC/LLC/SACF/FTC	15 (1.24%)	12 (5.6%)	3 (11.5%)	
**Clinical Frailty Score**	*n* = 233	*n* = 209	*n* = 24	** < *0.001***
▪ Non-frail (score 1–4)	181 (77.7%)	169 (80.9%)	12 (50.0%)	
▪ Frail (score 5–9)	52 (22.3%)	40 (19.1%)	12 (50.0%)	
**Katz ADL score (baseline)**	*n* = 211	*n* = 188	*n* = 23	***0.009***
▪ Fully independent (score 6/6)	171 (81.0%)	157 (83.5%)	14 (60.9%)	
▪ Not fully independent (score < 6/6)	40 (19.0%)	31 (16.5%)	9 (39.1%)	

*ACCI* age-adjusted CCI, *ADL* activities of daily living, *CCI* Charlson Comorbidity Index, *ERAS* enhanced recovery after surgery, *FTC* full-time carer, *HLC* high-level care, *IQR* interquartile range, *LLC* low-level care, *SACF* skilled aged care facility, *SD* standard deviation

Bold and italics = those the results that are statistically significant

Most patients (93.8%) were from home with or without family support prior to their colorectal admission. This was also consistent in both elective (94.4%) and emergency (88.5%) cohorts (Table 1). Overall, most patients who underwent a CRC operation were non-frail (77.7%); however, there was a significant difference between the elective (80.9%) and emergency (50.0%) cohorts (*p* < 0.001). Most patients aged ≥ 80 were functionally independent (81.0%) pre-operatively. A larger proportion of those who had emergency operations were not functionally independent (39.1%) compared to elective patients (16.5%) (*p* = 0.009). Of those who were not fully independent (*n* = 40), five were fully dependent. All five had a CFS of 7, ‘Living with severe frailty’, and three of these were elective patients. Two were at home with their spouse, who were their full-time carer.

### Primary outcome

#### Non-home discharge

Of the original 242 patients, 234 patients were analysed for discharge disposition and non-home discharge analysis (Table 2). Eight patients were excluded from the original cohort. There were seven (2.89%) deaths. One patient had spent extensive time in rehabilitation for sequential hip and duodenal ulcer operations. They were transferred for an ‘elective’ CRC resection and, post-operation, was deemed fit enough to go directly home, thus was excluded from NHD analysis due to a very unusual pre- and post-operative course. Overall, 44/221 (19.9%) of patients from home had a non-home discharge.

**Table 2:** Baseline, functional and operative factors of patients considered for NHD analysis (*n* = 234), and their association with NHD

	Total (*n*= 234)	Home discharge	Non-home discharge	*p*-value
		(*n* = 190)	(*n* = 44)	
**Baseline factors**				
Age (± SD)	85.1 ± 3.74	84.8 ± 3.54	86.2 ± 3.89	***0.02***
Male (%)	126 (53.8%)	108 (56.8%)	18 (40.9%)	0.056
Ex-smoker/smoker (%)	87 (37.2%)	71 (37.4%)	16 (36.4%)	0.901
CCI (median [IQR])	4.0 [3.0–6.0]	4.0 [3.0–6.0]	5.0 [3.0–7.0]	***0.39***
ACCI (median [IQR])	8.0 [7.0–10.0]	8.0 [7.0–10.0]	9.0 [8.0–11.75]	***0.02***
Admission acuity (emergency)	23 (9.8%)	13 (6.8%)	10 (22.7%)	***0.001***
**Functional factors**				
Clinical Frailty Score	*n* = 226	*n* = 184	*n* = 42	
Non-frail (1–4)	178 (78.8%)	150 (81.5%)	28 (66.7%)	***0.034***
Frail (5–9)	48 (21.2%)	34 (18.5%)	14 (33.3%)	
Katz ADL score (baseline)	*n* = 205	*n* = 155	*n* = 40	
Fully independent (6/6)	168 (82.0%)	142 (86.1%)	26 (65.0%)	***0.002***
Not fully independent (< 6/6)	37 (18.0%)	23 (13.9%)	14 (35.0%)	
ERAS status	*n* = 234	*n* = 190	*n* = 44	
ERAS pathway	164 (70.1%)	145 (76.3%)	19 (43.2%)	** < *0.001***
Non-ERAS pathway	70 (29.9%)	45 (23.7%)	25 (58.8%)	
**Operative factors**				
Adhesiolysis (vs no adhesions)	72 (30.8%)	59 (31.1%)	13 (29.5%)	*0.845*
Obstructed cancer	23 (9.8%)	13 (6.8%)	10 (22.7%)	***0.001***
Operation ended as laparotomy	70 (29.9%)	45 (23.7%)	25 (56.8%)	** < *0.001***
Extended resection: > 1 CR cancer	8 (3.4%)	6 (3.2%)	2 (4.5%)	*0.648*
Multi-visceral involvement	17 (7.3%)	14 (7.4%)	3 (6.8%)	*0.899*
Unexpected bleeding—agents/sutures	13 (5.6%)	11 (5.8%)	2 (4.5%)	*0.745*
Iatrogenic injury	18 (7.7%)	13 (6.8%)	5 (11.4%)	*0.310*
Formation of stoma	53 (22.6%)	39 (20.5%)	14 (31.8%)	0.107

Eight patients excluded from non-home discharge analysis: 1 patient came from rehabilitation and went home, and there were 7 deaths.*ACCI* age-related Charlson Comorbidity Index, *ADL* activities of daily living, *CCI* Charlson Comorbidity Index, *ERAS* enhanced recovery after surgery, *HD* home discharge, *IQR* interquartile range, *NHD* non-home discharge, *SD* standard deviation

Bold and Italic = results that are statistically significant

The nonagenarian cohort had a higher NHD rate (36.0%) compared to the octogenarian cohort (16.7%), *p* = 0.020. Emergency CRC patients aged ≥ 80 were more likely to have a NHD, OR 4.01 (95% CI 1.62–9.87, *p* < 0.001) compared to elective patients (43.5% vs 16.1%, respectively) (Table 3). There was no association between stoma formation and NHD.

**Table 3:** Overall cohort (*n* = 242): primary and secondary outcomes within the octo- and nonagenarian colorectal cancer cohort, stratified by elective and emergency cases

		**Admission acuity**		
	**Overall** **(*n* = 242)**	**Elective** **(*n* = 216)**	**Emergency** **(*n* = 26)**	***p*-value**
**Primary outcome:**	*n* = 234	*n* = 211	*n* = 23	** < *0.001***
‘Home’ discharge	190 (81.2%)	177 (83.9%)	13 (56.5%)	
Non-home discharge	44 (18.8%)	34 (16.1%)	10 (43.5%)	
Secondary outcomes				
Functional decline	*n* = 177	*n* = 159	*n* = 18	*0.06*
▪ No functional decline	131 (74.0%)	121 (76.1%)	10 (55.6%)	
▪ Functional decline	46 (26.0%)	38 (23.9%)	8 (44.4%)	
Discharge disposition	*n* = 242	*n* = 216	*n* = 26	** < *0.001***
▪ Independent living (± family)	178 (73.6%)	167 (77.3%)	11 (42.3%)	
▪ Inpatient geriatric/rehabilitation unit	43 (17.8%)	35 (16.2%)	8 (30.8%)	
▪ Nursing home (LLC/HLC/FTC)	14 (5.79%)	10 (4.62%)	4 (15.3%)	
▪ Death	7 (2.89%)	4 (1.85%)	3 (11.5%)	
Severe complications (CD III and IV)	20 (8.30%)	18 (8.33%)	2 (7.69%)	0.973
Length of stay (days), mean ± SD	10.1 ± 7.91	9.71 ± 7.97	13.4 ± 6.71	** < *0.001***

8 patients excluded from non-home discharge analysis: 1 patient came from a rehabilitation facility and went home after their operation, and there were 7 deaths

*CD* Clavien–Dindo, *FTC* full-time carer at home, *HLC* high-level care nursing home, *LLC* low-level care nursing home, *SD* standard deviation

Bold and italic = the results that are statistically significant

### Univariable and multivariable logistic regression analysis

All emergency patients had obstructing cancers. Of those, one also had a localised perforation. There were no hemodynamically unstable bleeding patients. Thus, admission acuity and emergency obstructed cancers had a perfect correlation and only admission acuity was used for the initial regression model.

Baseline Katz ADL status and CFS were strongly correlated (*r* =  − 0.752, *p* < 0.001), which was expected given they are both markers of premorbid functional status. Katz ADL had larger number of missing values, so only CFS was included in the final analysis. CCI and ACCI were strongly correlated (*r* = 0.990, *p* < 0.001) as expected. ACCI incorporated both, and thus was the only one used to represent both age and CCI in the regression analysis.

All baseline, functional and operative factors that were significant after adjustment for collinearity, confounding and effect modification were considered for multivariable analysis (Table 4). The remaining variables achieving statistical significance of *p* < 0.05 were used in the multivariable regression analysis.

**Table 4:** Univariable and multivariable regression analysis—only those pre-morbidly from home

**Outcome = non-home discharge**	***N* included**	**% Missing data**	**Odds ratio (95% CI)**	***p*-value**
**Initial variables considered for analysis**				
Baseline covariates				
Non-ERAS pathway	221	0	4.23 (2.12–8.43)	< 0.001
Clinical Frailty Score (5–9)	213	3.62%	3.06 (1.42–6.63)	0.005
Baseline Katz ADL	196	11.3%	4.72 (2.05–10.8)	< 0.001
CD complication III or IV	221	0	4.91 (1.90–12.7)	< 0.001
Age	221	0	1.12 (1.02–1.22)	0.014
Non-age-adjusted CCI	221	0	1.14 (1.00–1.30)	0.048
Age-adjusted CCI	221	0	1.16 (1.02–1.32)	0.022
Admission acuity (emergency)	221	0	4.44 (1.75–11.3)	0.002
Operative covariates				
Significant pathological adhesions	221	0	0.96 (0.46–1.97)	0.901
Invasion into other organs	221	0	0.85 (0.23–3.10)	0.808
Emergency obstructed cancer	221	0	4.44 (1.75–11.3)	0.002
Conversion to open/open operation	221	0	4.23 (2.12–8.43)	< 0.001
Unexpected bleeding requiring haemostatic agents or sutures	221	0	0.72 (0.15–3.36)	0.675
Synchronous resection	221	0	1.36 (0.26–6.97)	0.714
Other visceral injury	221	0	1.76 (0.59–5.30)	0.312
Formation of stoma	221	0	1.89 (0.91–3.95)	0.088
**Multivariable logistic regression**				
Clinical Frailty Score	213	0	2.91 (1.25–6.75)	0.013
Complication—CD III or IV	213	0	3.92 (1.40–11.0)	0.009
Conversion to open/open operation	213	0	3.93 (1.87–8.24)	< 0.001

*ADL* activities of daily living, *CCI* Charlson Comorbidity Index, *CD* Clavien–Dindo, *ERAS* enhanced recovery after surgery.

Of the entire cohort, 221 patients were included for final regression analysis for NHD after removing those who died, those from skilled aged care facility and the one patient that went from rehabilitation to home. In total, 44 patients were discharged to a skilled nursing facility, resulting in a NHD rate of 19.9%. The initial model used seven variables: non-ERAS pathway, complications, ACCI, admission acuity, CFS, conversion to open and requirement of stoma. After backward Wald elimination method, only three were included in the final model: Clinical Frailty Score, severe complications and conversion to open. Various models were run and tested by grouping ordinal variables and dichotomised functional data as part of sensitivity analysis.

In the multivariable analysis from those only originally from home, open/conversion to open operation (OR 3.93, 95% CI 1.87–8.24, *p* ≤ 0.001), severe complications (OR 3.92, 95% CI 1.40–11.0, *p* = 0.009) and Clinical Frailty Score (OR 2.91, 1.25–6.75, *p* = 0.013) remained significant after adjusting for all other variables.

The final model was well calibrated when assessing goodness of fit ($\chi$ ^2(4)^=2.083, *p* = 0.555) and had acceptable discrimination (*c*-statistic = 0.744 [95% CI 0.657–830]).

### Secondary outcomes

#### Functional decline

In total, there were 187 patients who had a post-operative ADL status recorded. Of these, 175 had both pre- and post-operative ADL status available to compare in the overall group and the group specifically that had a non-home discharge outcome. In the overall cohort, 131 (74.0%) returned to their baseline ADL status. Functional decline was recorded in 25.7% of patients in the overall cohort (*p* < 0.001). This was higher in the emergency cohort (44.4%), compared to the elective cohort (23.9%), *p* = 0.06.

Those who had functional decline were more likely to have a non-home discharge (72.4%) outcome in both overall and subgroup analysis (Table 5). Those who were not fully independent in their pre-admission Katz ADL score were also more likely to be frail, OR 82.4 (95% CI 28.5–238.3, *p* < 0.001), and had a higher rate of NHD, OR 3.32 (95% CI 1.52–7.23, *p* = 0.002). There was no difference in the rate of stoma formation and functional decline.

**Table 5:** Secondary outcomes and their relationship with NHD

	OVERALL				IN OWN HOME PRE-OPERATIVELY			
		Home discharge	Non-home discharge	***p***-value		Home discharge	Non-home discharge	***p***-value
Functional status change	*n* = 175	*n* = 146	*n* = 29		*n* = 167	*n* = 138	*n* = 29	
Functional decline	45 (25.7%)	24 (16.4%)	21 (72.4%)	** < *0.001***	44 (26.3%)	23 (16.7%)	21 (72.4%)	** < *0.001***
No functional decline	130 (74.3%)	122 (83.6%)	8 (27.6%)		123 (73.6%)	115 (83.3%)	8 (27.6%)	
Severe complications (CD III and IV)	20 (8.55%)	10 (5.26%)	10 (22.7%)	** < *0.001***				
Length of stay (days), mean ± SD	10.19 ± 7.97	8.81 ± 6.42	16.45 ± 10.91	** < 0.001**				

8 patients excluded from non-home discharge analysis: 1 patient came from rehab and went home, and there were 7 deaths.*CD* Clavien–Dindo, *HD* home discharge, *NHD* non-home discharge, *SD* standard deviation

Bold and Italic = results that are statistically significant

### Discharge disposition

The majority of patients (73.6%) were discharged home independently with family or with community-based help at home. A greater proportion of elective patients (77.3%) were discharged home (77.3%) compared to emergency patients (42.3%), *p* < 0.001.

Nineteen of the 44 (43.2%) NHD patients were transferred to a geriatric or medical unit within the same hospital prior to eventual discharge. Twelve of the 19 (63.2%) were eventually discharged to their own home with or without family support.

### Length of stay

The overall length of stay (LOS) was 10.1 ± 7.91 days in the overall cohort. The LOS was 3.69 days (*p* < 0.001) shorter in the elective group compared to the emergency group.

## Discussion

This is the first study to date to specifically investigate the rate and factors associated with non-home discharge in patients aged > 80 years undergoing CRC resection. Our study demonstrates a NHD rate of 19.9% in those patients aged ≥ 80 and pre-operatively from home living independently. Severe complications, frailty and open operations were significantly associated with non-home discharge in this subgroup analysis.

Discharge disposition nomenclature is slightly varied depending on the country and health service. ‘Skilled nursing facilities’, as described in studies from the USA, are synonymous with aged care facilities in Australia. Kanters et al. conducted a similar colorectal study in an American population and obtained their discharge data from NSQIP databases. It focused specifically on comparing academic and non-academic hospitals [17]. Their study differed to ours in their NHD definition. They used discharge disposition alone and did not compare this to pre-admission living arrangements. It potentially included patients in their NHD who may have originally come from a skilled nursing facility who eventually returned to the same. This population has been categorised as ‘home’ discharge in our study, and as such their 11.5% NHD rate potentially could be an overestimate. Their study otherwise is consistent with our findings that emergency admissions and those with severe complications have a higher rate of NHD [17]. However, the overall mean age was 63.3 years, and this study did not specifically look at the ≥ 80 age group, nor was it specific for colorectal cancer and included other colorectal pathologies.

Burguete et al. looked at NHD in those with cytoreductive surgery and hyperthermic intraperitoneal chemotherapy (HIPEC), inclusive of gastric, colorectal, primary peritoneal and ovarian cancers in a US-based population sourced from NSQIP data [18]. Their overall NHD rate was 7.9%; however, when looking specifically at their 136 colorectal cases, the rate was 9.7% [18]. The NHD population in their study was younger (mean age was 61 ± 21.6 years). Similarly to Kanters et al., they did not compare to pre-operative living situation prior to patient admission. This is real-world pragmatic selection bias as elderly, frail, functionally dependent and comorbid patients likely would not have been offered this significantly more morbid operation. It could potentially explain the reason for low NHD rates in their study in addition to a younger, less frail and comorbid cohort. Moreover, our centre does not perform cytoreductive surgery and HIPEC, and thus we could not compare this sub-cohort of patients. Two further US-based population studies have found NHD rates in colorectal cancer patients of 20.1–23.9% [19, 20]. Kelley et al. had a median age of 78 whilst Abz-El Aziz et al. did a subgroup analysis for those > 80 years [19, 20]. Whilst the focus of both these papers was not specifically on NHD, the age and NHD rate are most consistent with the results of our study. This would be expected given higher frailty and less functional independence with an ageing population and validates the findings from our study.

Vascular, orthopaedic, cardiac and gynaecological surgery have also investigated NHD as an outcome in their cohorts. Arya et al. found a 7.4% rate of NHD in a vascular cohort, with an overall mean age of 69.7. The mean age specifically for those with NHD was 73.9 years [21]. Penn et al. conducted a similar population-based study looking at NHD in patients who underwent surgery for gynaecological malignancies. They had a younger cohort, with a mean age of 59.7 years and a NHD rate of 3.1% [22]. Whilst a different specialty, the recovery and post-operative course from an oophorectomy or hysterectomy, compared to a bowel resection, are different, and thus could largely explain the significant differences in NHD rates.

Of this younger gynaecological cohort with a NHD, 78.8% were functionally independent. Our cohort demonstrated 65.0% and 68.8% in the ≥ 80 overall and 80–90 subgroup population, respectively. This is similar to the data from Australian Bureau of Statistics (ABS), whereby approximately 70% of older Australians were deemed to have no profound or severe core activity limitation and 86% were living in their own private residence [23]. A recent meta-analysis has confirmed that baseline functional status is a predictor of NHD [24]. Our population is slightly skewed compared to ABS data as those patients who would not be appropriate or offered an operation would not be captured in our dataset. This concept of ‘careful patient selection’, in determining who will survive with a good survival outcome and quality of life from an operation, is a widely established practice in surgery [25].

Our study is consistent with existing literature in gynaecological surgery demonstrating an association with laparotomies/open procedures and NHD [22]. Frailty is increasingly being used in the perioperative assessment of elderly CRC patients and has been shown to be associated with worse clinical and functional outcomes [14]. We found that patients who were frailer had a higher odds of having a NHD. Studies in emergency general surgery and gastric cancer specifically have demonstrated a similar association of frailty to NHD [21, 26–28]. No studies specific to colorectal cancer looking at these two variables have been conducted in the ≥ 80 population.

Overall, the concept of functional outcomes such as NHD is gaining significant traction in surgical and colorectal literature over the past decade. Ours is the first and only paper currently that sets out to analyse the frail, comorbid octo- and nonagenarian colorectal cancer population. In an older and frail colorectal cancer population, we found that almost one in five had a NHD, and we identified factors potentially contributing to this. This is also the first paper to specifically report NHD in the ≥ 80-year-old CRC population in existing literature.

## Limitations

This is a retrospective study, so no causal inferences can be made. Elderly patients not deemed appropriate for an operation would not be included in this dataset, thus already skewing the data to the more functionally and medically fit ≥ 80 CRC population. The authors appreciate that the study also does not look specifically at the extent or type of surgery (e.g. laparoscopic right hemicolectomy compared with an open TME resection). As an initial observational study, we investigated the rate of NHD in the overall older colorectal cancer surgery population. Future studies can specifically look into the particular types of resections. Interpreter bias of retrospective notes was accounted for by having two authors sample a selection of patients to obtain geriatric functional covariate data, one of whom was a geriatrician to improve the internal validity of the study.

## Conclusion

This is the first study in the literature that evaluates non-home discharge in the octogenarian and nonagenarian colorectal cancer population. It serves as a foundation to conduct future research into functional outcomes in this frail population, with two to three key contributing factors already being identified for further research. We found that almost one fifth of patients aged ≥ 80 have a non-home discharge outcome. The findings from this study are consistent with the few population-based US studies that have looked at this growing demographic, mostly as a subgroup analysis. Future prospective cohort studies exploring the factors identified in this study could be performed to determine causal relationships with non-home discharge.

## Data Availability

The data that support the findings of this study are available from the corresponding author upon reasonable request.
